# Supplementary figures and images for: Efficient and error-free fluorescent gene tagging in human organoids without double-strand DNA cleavage
Source: PLoS Biol. 2022 Jan 28;20(1):e3001527. doi: 10.1371/journal.pbio.3001527 (PMC8827455; doi:10.1371/journal.pbio.3001527)

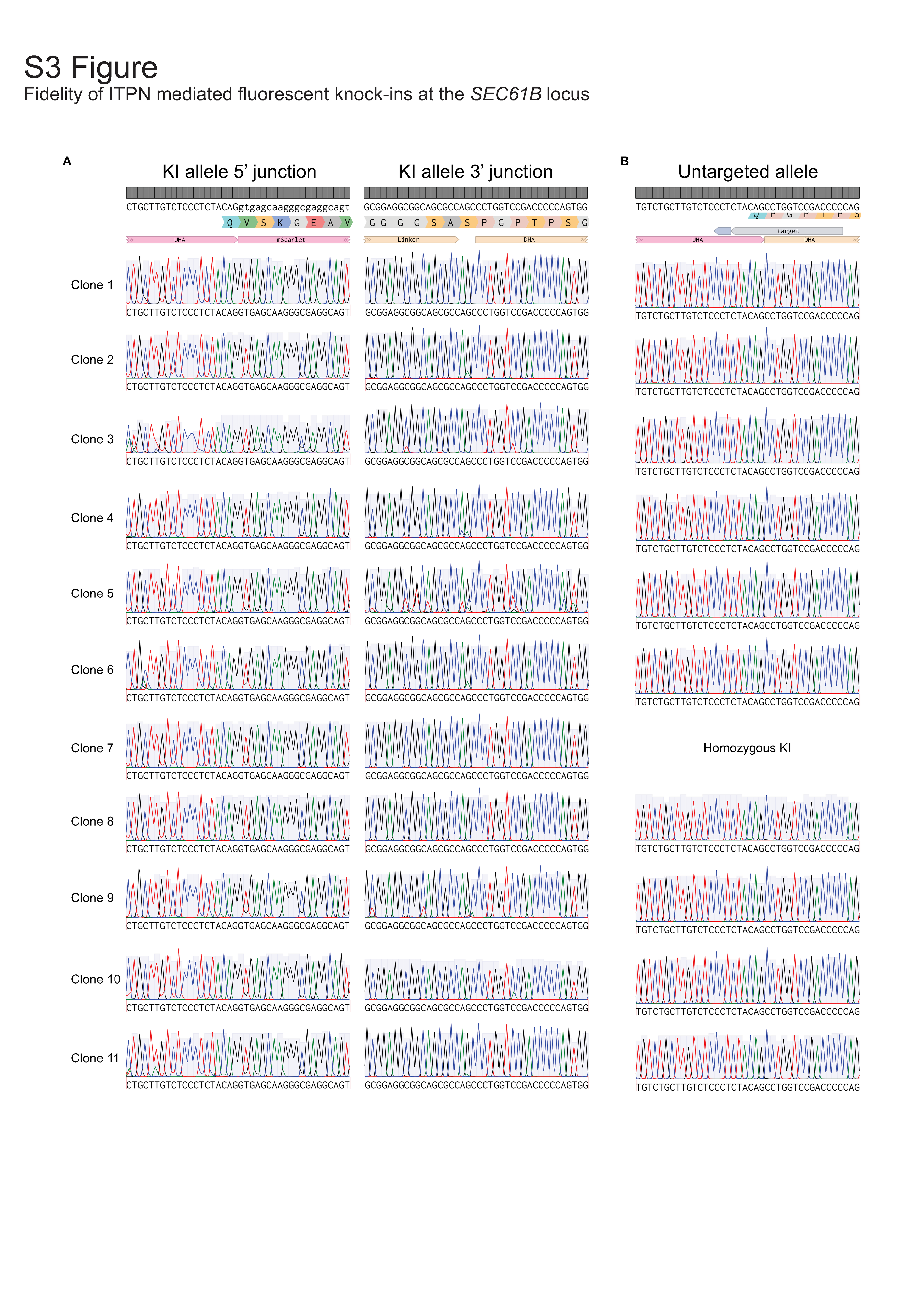

Supplement: S3 Fig — Sanger sequencing was performed on the SEC61B locus of 11 clonal patient-derived tumor organoid knock-in lines generated via ITPN (see Fig 1C). Knock-in lines were generated by handpicking individual large clonal knock-in organoids. The 5′ and 3′ junctions of the knock-in allele and the target region of the “untargeted” allele is shown for each clone. Clone no.7 carries a homozygous mScarlet knock-in at the SEC61B locus and therefore does not contain an untargeted allele. Underlying data are provided in S2 Data. ITPN, in-trans paired nicking. (TIF) [file pbio.3001527.s003.tif]

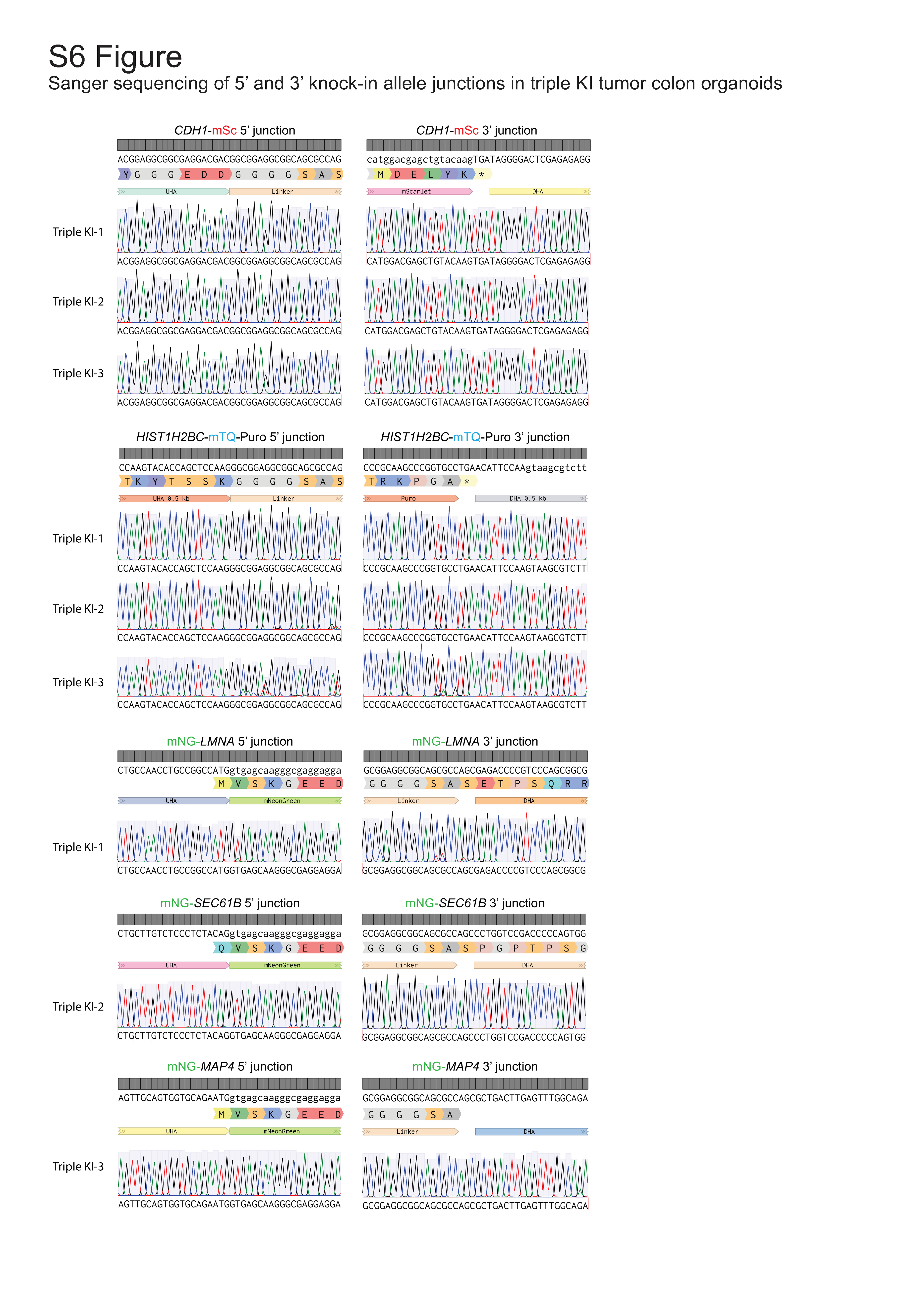

Supplement: S6 Fig — Sanger sequencing was performed on polyclonal triple knock-in tumor colon lines generated via manual picking of triple positive clonal organoids. The 5′ and 3′ junctions of each knock-in allele are shown for all 3 multiplexed knock-in conditions (see Fig 3). Underlying data are provided in S2 Data. (TIF) [file pbio.3001527.s006.tif]

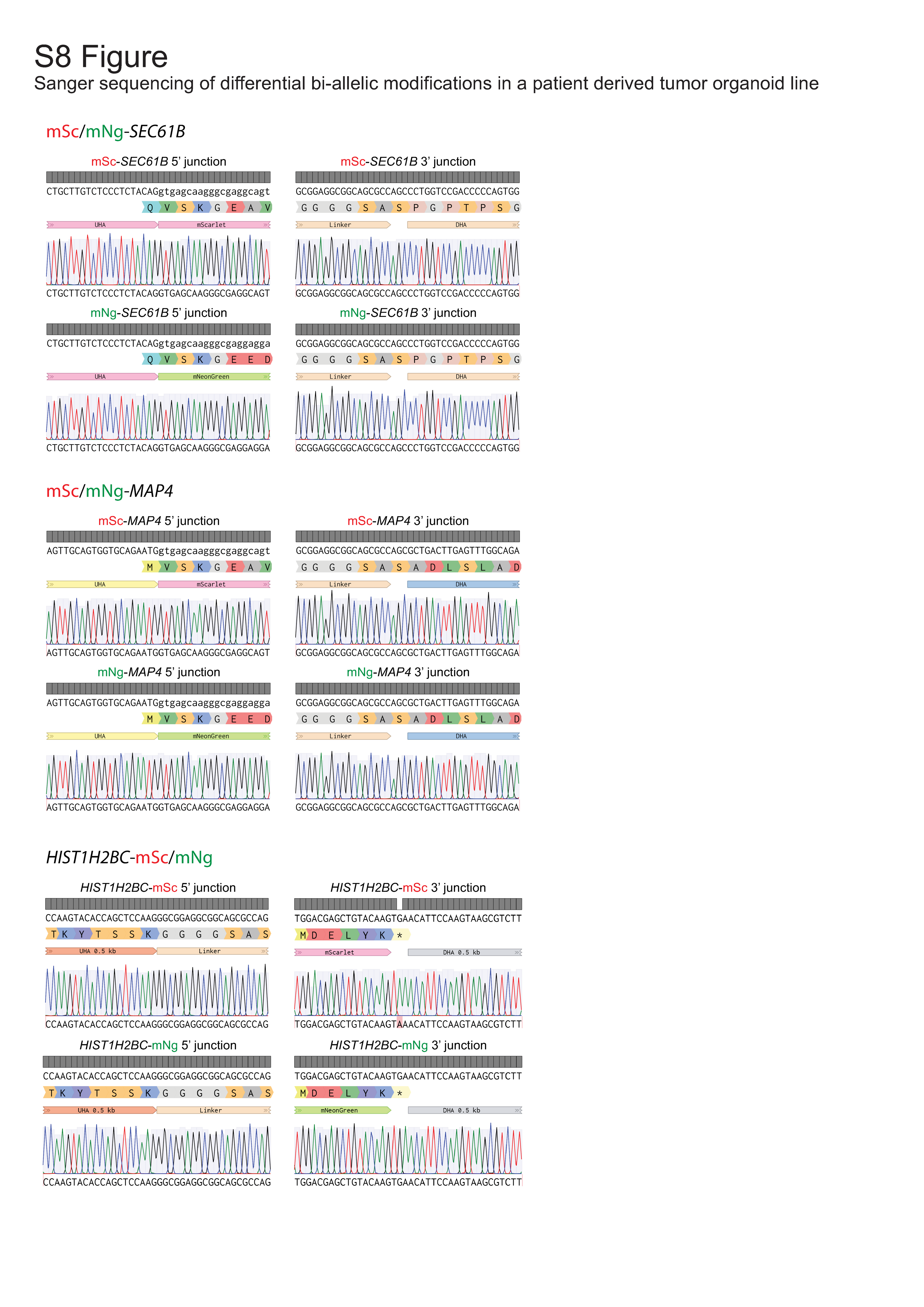

Supplement: S8 Fig — Sanger sequencing was performed on polyclonal biallelic tumor knock-in lines carrying both mScarlet and mNeongreen integrations at either the SEC61B, MAP4, or HIST1H2BC locus. The 5′ and 3′ junctions of each knock-in allele are shown for all 3 biallelic knock-in conditions (see Fig 4A). For HIST1H2BC-mSC, the endogenous stop codon of the HIST1H2BC locus was maintained. (TIF) [file pbio.3001527.s008.tif]
