# Supplementary material for: Efficient and error-free fluorescent gene tagging in human organoids without double-strand DNA cleavage
Source: PLoS Biol. 2022 Jan 28;20(1):e3001527. doi: 10.1371/journal.pbio.3001527 (PMC8827455; doi:10.1371/journal.pbio.3001527)
Supplement: S1 File — (DOCX) [file pbio.3001527.s015.docx]

**S1 File**

**Design considerations and cloning protocol for the one-step targeting vector assembly to obtain fluorescent gene tagging using in-trans paired nicking or cleavage (Bollen & Hageman et al., PLOS Biology).**

1. **Considerations regarding fluorescent gene tagging**

Before starting the design and cloning of a targeting vector to obtain fluorescent knock-ins for the purposes of (live) cell imaging of endogenous proteins the researcher should consider the following:

1. *Is the protein of interest expressed at sufficient levels to support the intended application?* Endogenous expression levels of proteins are generally at least an order of magnitude lower when compared to exogenous over-expression. This can complicate live cell imaging procedures, since the lower expression levels require more light exposure to produce sufficient signal, which in turn accelerates bleaching and laser toxicity. In our experience, highly expressed proteins such as H2B1C can be imaged dynamically (4-minute intervals) on confocal systems over a period of at least 72h.
2. *Which terminus should be targeted for fluorescent gene tagging?* We recommend a pre-study of current literature on the behavior and correct sub-cellular localization of fluorescently-tagged exogenous over-expression of the protein of interest, to infer whether the protein of interest can be fused without compromising its activity. Often, the least invasive terminus can be rationally deduced. For example, membrane proteins frequently have a terminus embedded in the membrane or exposed to the cell exterior, which is not suitable for fluorescent tagging.
3. *How will the cells carrying the fluorescent knock-in be selected for?* Generally, if a protein of interest is suitable for live cell imaging it will also be sufficiently bright to allow FACS of knock-in cells. There are exceptions. Some cell or organoid lines may not tolerate single cell outgrowth after FACS. Furthermore, the protein of interest may only be expressed in differentiated cells and not in the primary cells initially targeted. In the former case, we advocate using a C-terminal P2A-selection element when possible, which allows antibiotic enrichment of correctly targeted cells. If a C-terminal knock-in is not compatible or if the protein is expressed only in differentiated cells, resorting to a sampling-based selection of clonal lines may be the easiest option. Generally, knock-in efficiencies are sufficiently high to find a knock-in by sampling fewer than 40 clones. Alternatively, the researcher can modify the targeting vector backbone (S2 File) to include more complex and sophistigated reporter constructs like a floxed selection element that is expressed by an independent promoter^1^.
4. *Fluorescent protein choice.* For the targeting vector backbones described in this manuscript we selected fluorescent proteins that are bright and have a high monomeric propensity (with the exception of Dendra2 which was chosen for its photoconvertible properties). Monomeric property is especially important given that dimerization of endogenously tagged fluorescent proteins is associated with perturbed function and localization defects. For an overview of the characteristics of many fluorescent proteins we recommend the excellent work by Cranfill et al.^2^.
5. **Cas9 target site selection**

To discuss considerations for Cas9 target site selection we will use an N-terminal LMNA knock-in as a concrete example. We recommend using Benchling.com for knock-in designs, which is free for academics.

We first import the annotated human LMNA genomic sequence and find the site where we want to integrate our knock-in which, in the case of LMNA, is at the N-terminus, right after the start codon:


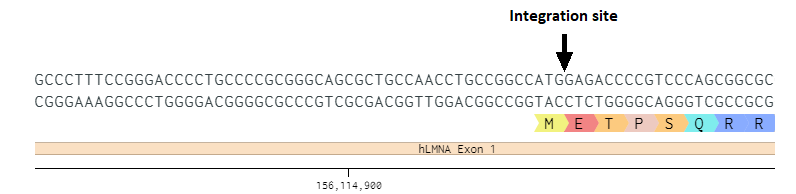


We then select a sequence spanning 50 bases up- and downstream of the integration site and scan for SpCas9 target sites. The image below represents the Benchling output, slightly modified for clarity. Outputs of other design tools generally look similar. There are plenty of SpCas9 target sites available, we highlighted a few to discuss target site selection considerations:


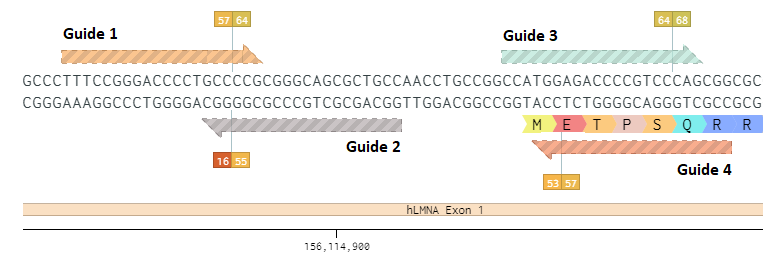


Each target site is represented by an arrow. The direction indicates whether the guide RNA anneals to the sense or antisense genomic strand. The cut site, 3-bp upstream of the PAM, is indicated by the flag pole for each guide. The first number of the flag represents the predicted on-target cleavage efficiency and the second number represents specificity. In each case, higher is better.

In our view, there are 4 considerations for target site selection in order of importance:

1. Cleavage proximity to the integration site
2. The Cas9-target site overlaps the integration site
3. Predicted on-target efficiency (>50 is great)
4. Specificity (not important when editing using in-trans paired nicking).

In the image above **guide 2** is the worst option, the cut is far away from the integration site and the on-target efficiency score is bad. **Guide 1** is slightly better as it has a good on-target efficiency score, but cuts equally far away from the integration site. **Guide 3** is much better; it has a great on-target efficiency score and cuts closer to the integration site. However, although **guide 3** overlaps the integration site, the integration of the knock-in still preserves 15-nucleotides of the SpCas9 target site including the PAM (the integration site is determined by the homology arms and not by the Cas9 cut, the cut serves solely to stimulate homology-directed repair). Leaving a too large a portion of the original target site intact risks re-targeting of the correctly edited allele by Cas9. Fore safety, we use a cut-off of 14nt’s of less^3^. Therefore, the use of **guide 3** (and indeed guide 1 & 2) would require editing of the homology arms to introduce silent mutations that prevents Cas9 cleavage after successful editing. In the case of **guide 3** for example; selecting a different codon for arginine (CGG -> CGC) in the targeting vector would already destroys the PAM. For **guides 1 & 2**, it is harder to introduce a non-invasive mutation since these guides cut in the 3’-UTR which risks disrupting important motifs. Therefore, in cases where we cannot select a guide that sufficiently overlaps the integration site such that re-cleavage by Cas9 is prevented, we have a preference for guides that cut in the coding region, since this allows the introduction of silent mutations that prevent re-cleavage (such as guide 3). Fortunately, **guide 4** allows a knock-in design where the introduction of silent mutations is not necessary. **Guide 4** has a good on-target score and cuts very close to the integration site (1bp downstream), in addition, the target site is destroyed by the introduction of our knock-in. For these reasons we selected **guide 4** for our design.

With respect to specificity, we strongly recommend performing the knock-in with a single genomic nick instead of double-strand cleavage, such that potential indels at off-target sites can be disregarded. If the use of wild-type Cas9 is desired, we still recommend to disregard the score indicated by the flag. Instead, we suggest taking a look at the top predicted off-target sites for each guide to check if they are located in genes. If they are, there is no reason to discard the guide. Instead, when using wild-type Cas9 for editing, the positions of the off-target sites can be sequenced for individual clones to confirm the absence of indels at these sites (unless the off-target site is a 100% match, resulting a very high indel frequency).

While we think the predicted on-target cleavage efficiency is important, we generally do not discard well positioned target sites with mediocre on-target scores in favor of target sites with great on-target scores that cut farther away from the integration site. For example, the target sites selected for the H2B1C knock-in in our manuscript has a poor predicted on-target score (16.3) but a great cleavage position, and results in some of the highest knock-in efficiencies we have recorded.

The strategies outlined above are also relevant for alternative CRISPR associated nucleases, in particular with respect to the the Cas9 derived nucleases with alternative PAMs.

1. **Homology arm design for one-step targeting vector assembly for N-terminal knock-ins.**

To integrate the coding sequence of a fluorescent protein at a specific genomic location, we select up- and downstream homology arms that flank the desired integration site. In the case of LMNA, we integrate our knock-in right after the start codon at the N-terminus.

Homology arm length is a frequently debated topic. Prior work^4-6^, and our experiments in Fig 1D of the current manuscript, lead us to operate with a default homology arm length of 600bp. This is a convenient size for commercial synthesis or a genomic PCR, and generally results in high knock-in efficiencies for both in-trans paired cleavage and nicking. We first select our homology arms for LMNA:


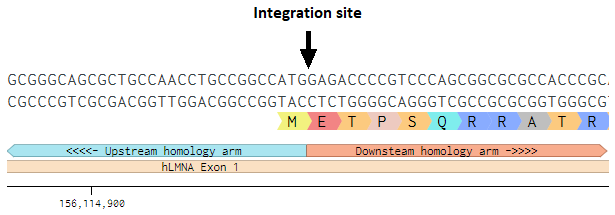


To support in-trans paired cleavage and nicking, we prefer to flank both homology arms by the same Cas9-target site as the one selected for genomic cleavage at the integration site, so that co-delivery of a single guide is sufficient, instead of using a separate, dedicated guide for vector cleavage. On the other hand, a dedicated vector guide allows the researcher to select a well vetted and highly efficient guide for vector cleavage, but requires co-delivery. Both strategies are valid. Furthermore, we designed our targeting vector backbones to allow one-step targeting vector assembly using a SapI based golden-gate strategy, which requires flanking the homology arm fragments with SapI sites that generate specific overhangs.

To generate homology arm fragments that are flanked with a Cas9 target site and SapI restriction sites, we prefer commercial synthesis. We created homology arm fragment templates that can be modified and ordered. Note that the N-terminal and C-terminal targeting vector backbones require different overhangs. Here are the templates for the N-terminal targeting vectors:

**N-terminal Upstream Homology arm (UHA) fragment template**

AACGCTCTTCATAC-Cas9 target here-PAM-UHA here-GTGTGAAGAGCGCG

**N-terminal DHA fragment template**

CGCGCTCTTCGAGC-DHA here-Cas9 target here-PAM-AATCGAAGAGCGTT

In the above homology arm fragment templates, the SapI sites are indicated in green. These sites generate unique 3-base overhangs that allow golden-gate assembly of the targeting vector.

SapI restriction enzyme

5'...GCTCTTC (N)_1_... 3'

3'...CGAGAAG (N)_4_... 5'

The selected homology arm regions can simply be copied and pasted, 3’ to 5’ to replace “UHA/DHA here”. If necessary, include mutations in the homology arms that prevent re-cleavage by Cas9. The Cas9 target site, **including the PAM** can also simply be copied and pasted from the genome. The respective orientation of the Cas9 target sites when using in-trans paired nicking is irrelevant (e.g., it makes no difference whether you nick the top-strand twice, or both the top and bottom strand once).

Before ordering the fragments, we strongly encourage in-sillico golden-gate assembly (Benchling, or any other cloning program) using the desired targeting vector backbone. This confirms that the SapI overhangs are correct and allows the researcher to check if the ultimate targeting vector has the intended sequence upon vector assembly and upon insertion into the genome.

Alternatively, the homology arm fragments can be generated using a genomic PCR with the required overhangs. The primer templates for N-terminal assemblies are:

**N-terminal UHA fragment**

Forward 5’-3’:

AACGCTCTTCATAC-Cas9 target here **with PAM** -NNNNNNNNNNNNNNN

Reverse 5’-3’:

CGCGCTCTTCACAC-NNNNNNNNNNNNNNN

**N-terminal DHA fragment**

Forward 5’-3’:

CGCGCTCTTCGAGC-NNNNNNNNNNNNNNN

Reverse 5’-3’:

AACGCTCTTCGATT-Cas9 target here **with PAM** (reverse complement)-NNNNNNNNNNNNNNN

We strongly encourage performing an in-silico PCR and subsequent in-silico golden-gate assembly to confirm that the targeting vector has the intended composition and that the Cas9-target sites are correct.

- 1. **Homology arm design for C-terminal knock-ins.**

For C-terminal fluorescent knock-ins the target site selection criteria can be applied as previously described. C-terminal fluorescent knock-ins are integrated right before the endogenous stop codon. However, since the C-terminal targeting vector backbones already contain a stop codon, the endogenous stop codon can be omitted from the downstream homology arm. Below is an example of the homology arm locations of our H2B1C knock-ins, omitting the stop codon:

**
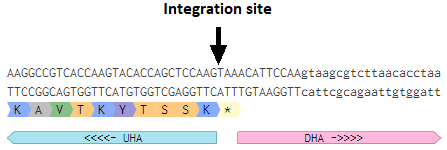
**

As for N-terminal knock-ins, the homology arms and target sites can be pasted into homology arm fragment templates for C-terminal targeting vector backbones as previously described:

**C-terminal UHA fragment template**

AACGCTCTTCATAC-Cas9 target here-PAM-UHA here- GGCTGAAGAGCGCG

**C-terminal DHA fragment template**

CGCGCTCTTCGTGA-DHA here-Cas9 target here-PAM- AATCGAAGAGCGTT

If preferred, the homology arm fragments can be amplified from genomic DNA using the following primer templates:

**C-terminal UHA fragment**

Forward 5’-3’:

AACGCTCTTCATAC-Cas9 target here **with PAM**-NNNNNNNNNNNNNN

Reverse 5’-3’:

CGCGCTCTTCAGCC-NNNNNNNNNNNNN

**C-terminal DHA fragment**

Forward 5’-3’:

CGCGCTCTTCGTGA-NNNNNNNNNNNNNN

Reverse 5’-3’:

AACGCTCTTCGATT-Cas9 target here **with PAM** (reverse complement)-NNNNNNNNNNNNNNN

1. **One-step targeting vector assembly**

For the purposes of one-step golden gate assembly of targeting vectors, we assume the homology arm fragments have been gel purified (genomic PCR) or resuspended (commercial) to a concentration of ~10ng/ul.

**SapI based golden gate reaction mix:**

10x T4 ligation buffer 1.5μl

10mM ATP 1.5 μl

SapI 1 μl

T4 ligase 1 μl

TV backbone 100ng X μl

Upstream arm 10ng X μl

Downstream arm 10ng X μl

**MQ To 15ul**

**Thermocycler program**:

37˚C 2'

16˚C 5'

**25x**

---

37˚C 15'

80˚C 10'

4˚C Forever

After the golden gate reaction, the mix can be transformed into bacteria using Ampicillin selection. Generally, we miniprep 6 colonies per construct and run an restriction fragment length polymorphism assay to scan for correct clones.

Correct integration of the homology arms can subsequently be checked using the following sequencing primers, which are suitable for both N- and C-terminal targeting vectors:

UHA_seq: caaataggggttccgcgcac

DHA_seq: ttaccgcctttgagtgagctga

**Citations**

1. Bollen Y, Post J, Koo BK, Snippert HJG. How to create state-of-the-art genetic model systems: strategies for optimal CRISPR-mediated genome editing. *Nucleic Acids Res*. 2018;46(13):6435-6454. doi:10.1093/nar/gky571
2. Cranfill PJ, Sell BR, Baird MA, et al. Quantitative assessment of fluorescent proteins. *Nat Methods*. 2016;13(7):557-562. doi:10.1038/nmeth.3891
3. Fu Y, Sander JD, Reyon D, Cascio VM, Joung JK. Improving CRISPR-Cas nuclease specificity using truncated guide RNAs. *Nat Biotechnol*. 2014;32(3):279-284. doi:10.1038/nbt.2808
4. Chen X, Janssen JM, Liu J, et al. In trans paired nicking triggers seamless genome editing without double-stranded DNA cutting. *Nat Commun*. 2017;8(1):657. Published 2017 Sep 22. doi:10.1038/s41467-017-00687-1
5. Zhang JP, Li XL, Li GH, et al. Efficient precise knockin with a double cut HDR donor after CRISPR/Cas9-mediated double-stranded DNA cleavage. *Genome Biol*. 2017;18(1):35. Published 2017 Feb 20. doi:10.1186/s13059-017-1164-8
6. Yao X, Wang X, Hu X, et al. Homology-mediated end joining-based targeted integration using CRISPR/Cas9. *Cell Res*. 2017;27(6):801-814. doi:10.1038/cr.2017.76
