# Supplementary material for: Efficient and error-free fluorescent gene tagging in human organoids without double-strand DNA cleavage
Source: PLoS Biol. 2022 Jan 28;20(1):e3001527. doi: 10.1371/journal.pbio.3001527 (PMC8827455; doi:10.1371/journal.pbio.3001527)
Supplement: S2 File — (DOCX) [file pbio.3001527.s016.docx]

**S2 File**

**Personalize your one-step targeting vector backbone by replacing the current donor templates (Bollen & Hageman et al., PLOS Biology).**

The two major steps involved are: 1) design/order of your personalized donor template to be inserted into the targeting backbone. 2) Linearize the backbone of our existing targeting vectors.

**1. Replace donor templates to personalize your one-step targeting vector backbone**

Personalized targeting vector backbones with up-to-date fluorophores or more complex and sophisticated reporter constructs can easily be generated by modifying our existing templates for either N- or C-terminal knock-ins.

Personalized donor templates should be sequence-customized to be compatible with our seamless one-step assembly strategy of our targeting vector backbones. Please see the end of this document for the sequence information of vector backbones for the N and C-terminal knock-ins. In short, your reporter construct represents the [insert] and should be flanked with the DNA sequence as indicated. Fully customized donor templates can be ordered as a DNA fragment from commercial suppliers (e.g. ID&T or Genscript) and is suitable for Gibson assembly^1^ or recombinase based seamless cloning in combination with the PCR amplified vector backbone fragment.

General note: When modifying the SapI overhangs, ensure that each of the 4 overhangs carries at least two unique bases so that the golden gate assembly has a high ligation specificity. Before ordering the DNA fragment, we strongly encourage an in-sillico assembly of the personalized targeting vector backbone followed by a subsequent in-sillico integration of the desired homology arms to confirm that the cloning overhangs are compatible.

**2. Generating locus-specific targeting vectors**

Personalized targeting vector backbones can be generated by combining the desired insert, designed according to our insert template (described below), with a PCR amplified vector backbone.

To generate a linearized backbone fragment, use the following primers on one of our existing targeting vectors as a PCR template.

TVBB_FWD: tcctcgctcactgactcgct

TVBB_REV: gcggtattttctccttacgcatctg

After gel purification of the generic backbone fragment (1.9kb in length) and reconstitution of the commercially ordered personalized donor template, the new targeting vector backbone plasmids can be assembled using Gibson assembly or recombinase based cloning kits according to the manufacturer’s specifications. Once the new targeting vector backbone is finalized, the plasmid is ready for the insertion of both homology arms (see S1 File).

**Donor templates for N- and C-terminal targeting vector backbones**

**Donor template for N-terminal knock-ins**

**5'gcgtaaggagaaaataccgc**TACGGAAGAGCGCGATCGCGTTTAAAGGCTCTTCAGTG**[insert]**GGCGGAGGCGGCAGCGCC**AGC**CGAAGAGCGTTTAAACGCGAACCGGCTCTTCGAAT**tcctcgctcactgactcgct 3'**

- **Bold & underscored** are the 20nt Gibson/recombinase cloning overhangs compatible with the PCR amplified backbone fragment.
- SapI sites that allow seamless one-step integration of up-and downstream homology arms (see S1 File)

SapI restriction enzyme

5'...GCTCTTC (N)_1_... 3'

3'...CGAGAAG (N)_4_... 5'

- GTG encodes the amino acid valine and serves as a SapI overhang for seamless cloning. Valine is the first amino acid of many fluorescent protein’s and is meant to be the first codon after the endogenous start codon. When modified, also modify the corresponding SapI overhang in the homology arm fragment.
- **[insert]** This is the position to place your own desired reporter construct sequence e.g. a different fluorescent protein.
- The blue segment encodes a GGGGSAS flexible linker that is present in both N- and C-terminal backbones to create a linker sequence between the fluorescent protein and the protein of interest. If undesired the sequence can be removed. However, for N-terminal assemblies, the last codon of the linker sequence (bold and underscored) serves as a SapI overhang for seamless cloning and should be conserved unless modifying the corresponding SapI overhang in the homology arm fragment.

**Donor template for for C-terminal knock-ins**

5'**gcgtaaggagaaaataccgc**TACGGAAGAGCGCGATCGCGTTTAAAGGCTCTTCA**GGC**GGAGGCGGCAGCGCCAGC**[insert]TGA**CGAAGAGCGTTTAAACGCGAACCGGCTCTTCGAAT**tcctcgctcactgactcgct** 3'

- **Bold & underscored** are the 20nt Gibson/recombinase cloning overhangs compatible with the PCR amplified backbone fragment.
- SapI sites that allow seamless one-step integration of up- and downstream homology arms (see S1 File)

SapI restriction enzyme

5'...GCTCTTC (N)_1_... 3'

3'...CGAGAAG (N)_4_... 5'

- The blue segment encodes a GGGGSAS flexible linker that is present in both N- and C-terminal backbones to create a linker sequence between the fluorescent protein and the protein of interest. If undesired (for example when performing a knock-in as an expression readout by integrating a P2A-flurophore at the C-terminus) the sequence can be removed. However, for C-terminal assemblies, the first codon of the linker sequence (bold and underscored) serves as a SapI overhang for seamless cloning and should be conserved unless modifying the corresponding SapI overhang in the homology arm fragment.
- **[insert]** This is the position to place your own desired reporter construct e.g. a different fluorescent protein
- **TGA** encodes a stop codon and serves as a SapI overhang for seamless cloning. When modified, also modify the corresponding SapI overhang in the homology arm fragment.

**Citations**

1. Gibson DG, Young L, Chuang RY, Venter JC, Hutchison CA 3rd, Smith HO. Enzymatic assembly of DNA molecules up to several hundred kilobases. *Nat Methods*. 2009;6(5):343-345. doi:10.1038/nmeth.1318
