# Supplementary material for: Efficient and error-free fluorescent gene tagging in human organoids without double-strand DNA cleavage
Source: PLoS Biol. 2022 Jan 28;20(1):e3001527. doi: 10.1371/journal.pbio.3001527 (PMC8827455; doi:10.1371/journal.pbio.3001527)
Supplement: Attachment — Submitted filename: Rebuttal PLOS 211115.docx [file pbio.3001527.s017.docx]

**Reviewer #1**

*In this manuscript, Bollen et al reported a double-strand DNA-free system to allow seamless one-step targeting vector assembly to support both N- and C-terminal fluorescent knock-ins. Three genomic loci were simultaneously modified in a single round of targeting in colon cancer organoids. Similar approaches were applied on healthy colon organoids. This can be a potential very useful tool for organoid field. However, some concerns need to be addressed before considered for publication.*

Major concerns:

*1. The targeting efficiency needs to be better characterized. Based on Figure 3b. All cells in one organoid express the knock-in proteins. Is this true for all organoids? If yes, what is the percentage of organoids that are triple positive, what is the percentage of organoids that express two fluorescent proteins, what is the percentage of organoids that express mScarlet, mTurquoise2, or Neongreen?*

We thank the reviewer for critically assessing our manuscript. We apologize for unclarity and have improved our manuscript to avoid further misunderstandings regarding the exact nature of our knock-in procedures and its efficiency.

Regarding the procedure:

We now start the manuscript with a new schematic representation (Fig 1A) to clarify our workflow to generate knock-ins. Moreover, as depicted in Fig 1A, all our experiments now involve organoids including all the new data presented in Fig 1A. Briefly, prior to electroporation of organoids, we trypsinize the culture to obtain a cell suspension. The electroporated cells are newly seeded and can form organoids for 10 days during which the non-integrated vectors are cleared from the cells. Moreover, during this outgrowth period the absolute number of knock-in cells increases. Ultimately, we assess the knock-in efficiency within the organoid culture on day 10 by trypsinizing the entire population of electroporated organoids to a single cell suspension for subsequent flow analysis. In addition to measuring targeting efficiency, the fraction of knock-in cells can now be isolated using FACS to start a polyclonal knock-in line.

Regarding the organoid images, these are clonal knock-in organoids derived from single cells, hence the homogeneous expression pattern. We regularly observe a mosaic knock-in pattern in organoids when a cell within a tiny cell clump was edited. These negative cells can be depleted from the culture either by FACS purification, applying negative selection against these cells, or manual picking of secondary seeded clonal organoids.

Regarding the efficiency:

As explained above, the targeting efficiency is derived from the fraction of the knock-in cells that prior to any selection resides within the entire electroporated organoid culture. As expected, when performing multiplexed gene editing, the percentage of knock-in cells is dominated by cells carrying a single edited gene, with smaller fractions of cells carrying multiple knock-ins. We have now summarized all our targeting efficiencies in a new table (Fig 3A), depicting knock-in efficiency for each gene during the different triple targeting strategies, as well as their combinations. Please note, these efficiencies are prior to any selection.

*2. Is there any difference of targeting efficiency between normal and cancer organoids? The targeting efficiency should be quantified for both normal and cancer organoids as Question 1.*

We have now compared the knock-in efficiency of an N-terminal mScarlet knock-in at the *SEC61B* locus in side-by-side setting between normal and tumor organoids. Although the knock-in efficiency in normal organoids appeared to be slightly lower (although non-significant) than tumor organoids, these data showed that normal organoids can readily be modified with ITPN. The difference in efficacy is influenced by many variables, including differences in cell composition of the organoids, differences in electroporation efficiency and differences in organoid forming capacity of cells that received the plasmids. Foremost, the knock-in efficiency of our protocols for both tumor and normal organoids is sufficiently high because it results in a robust generation of knock-in lines.

To further corroborate the notion that normal organoids can be edited using ITPN, we have now generated an additional knock-in at the *KRT20* locus of normal human colon organoids (S4 Fig). KRT20 is a marker for differentiated epithelial cells of the colon. These organoids display the expected subcellular localization and also accurately show differences in expression levels between cells. We would also like to inform the referees with more recent data from our lab where various types of knock-in reporters have been integrated in the *LGR5* locus of normal human organoids (rebuttal Fig 1, for reviewers/editor only). Moreover, these reporters are >3kb in length, indicative that also complex multipurpose reporters are compatible with ITPN-mediated knock-ins. Lastly, the (nuclear) fluorescent pattern by these LGR5 knock-ins matches the expected localization of LGR5 stem cells at the entire base of crypt structures, including its downregulation once these stem cells start to differentiate and migrate away from crypt bottoms.

We are preparing a new research manuscript that will center around the creation and application of these new *LGR5* knock-ins. We hope that the referee will understand our reasoning not to include these *LGR5* knock-ins in the current manuscript, as their exact nature and application will not be of additive value to this technical manuscript where we state that ITPN enables efficient, error-free creation of knock-ins in human organoids.

**For reviewers/editor only:**

**Rebuttal Fig 1. LGR5 knock-in in normal colon organoids.**

*3. Figure S2C, sequencing of isolated clones should be performed to validate the heterozygous KI.*

We regretfully acknowledge that we underperformed on the sequence validation of our knock-in clones in the original manuscript, as was also correctly pointed out by referee #3. In the revised manuscript, we now provide extensive sequence validation of our knock-in clones that were generated in Fig 1, as well as the multiplex knock-ins from Figs 3 and 4. For more elaborate explanations, please see our rebuttal to the comments of reviewer #3.

Minor concerns:

*1. Scale bars are missing for most figure panels, which should be added and described in the legends.*

We apologize for this mistake. We have now added scale bars to all imaging panels.

*2. Gating control should be added for all flow cytometry analysis.*

We have recorded most data using gating controls (although the negative cell population acts as an internal gating control). The control data was added to the bar graphs in Fig 1.

We apologize to the reviewer for the omission of these control data. We now show flow cytometry plots of knock-in conditions and their controls in the new S1 Fig.

*3. Statistical analysis is missing in Figure 1e.*

Our sincere apologies. First of all, we have replaced all panels of the original Fig 1 with new experiments that now only involve organoid-related data (see also comments referee #2). Second, all experiments are now performed n=3 or >3, error bars are added to the graphs, as well as statistical analysis when informative.

**Reviewer #2**

[identifies herself as Joo-Hyeon Lee]

Summary

*Efficient CRISPR based gene targeting and generation of knock-ins have been challenging for 3D organoids derived from primary tissue. This manuscript describes a method to generate fluorescent reporter organoid lines by introducing homology directed repair (HDR) using Cas9 D10A nickase, which instead of creating a double stranded break (DSB) nicks only the allele targeted by the gRNA. The protocol would be beneficial to the field for efficient generation of human organoid reporter lines enabling the long-term tracing of cells. The authors showed the way to knock-in fluorescent proteins into multiple locus of the same cells, and further generate homologues lines by sequential targeting of both gene alleles. The classical antibiotics-based selection strategy could also be used in conjunction with florescence. These various plasmids used for fluorescent knock-in are also available with different fluorescent proteins to the community, proving that this protocol can be broadly useful.*

General Comments

*Originality and significance: The strategy of in-trans paired nicking using modified Cas9 nicking variant has been reported in 2D culture systems such as HEK293T cells, as cited in the manuscript (Chen et al., 2017). This protocol adapted the in-trans paired nicking strategy to generate florescence-based selection in primary tissue-derived organoids, which can also be combined with antibiotics-based selection, allowing the efficient genetic engineering of primary human organoid culture models.*

*Data and methodology: A major concern in this manuscript is that there is no description of biological replicates and validations in Fig. 3 and 4. For this system to be widely applicable, the authors should test multiple 'colonic' organoids and provide the detailed information of variation and efficiency of this strategy across biological repeats.*

*Conclusions: The authors used HEK293T cells to calculate the editing efficiency of their strategy in Fig. 1. Given that this manuscript focuses on 3D human primary organoids, the editing efficiency of in-trans paired/cleavage method should be provided in human primary organoid culture systems with appropriate statistics.*

Suggested improvements:

*1. Fig. 1: As stated above, it is crucial to provide the editing efficiency and the determination of homology arm length in human primary organoids, instead of HEK293T cells. Further, in Fig. 1b, did the authors observe the off-target effects with WT Cas9? TIDE analysis with sanger sequencing data would be useful to determine off-target effects in both WT Cas9 and nickase Cas9. In Fig. 1c, homozygous knock-in by FACS analysis must be supported by locus sequencing data either in the figure or as supplementary dataset.*

We thank the reviewer for critical reading of our manuscript and the great suggestions to improve our manuscript, starting with the notion to replace all our data regarding editing efficiencies of HEK293T cells with organoid data. We took these comments to heart and adapted the manuscript accordingly.

In the revised manuscript, we now characterize and compare the editing efficiency of various knock-in strategies in human organoids (in new Fig 1), followed by a TIDE and Sanger sequencing analysis (S2-3 Figs) that highlights the fidelity of in-trans paired nicking when compared to the use of wild-type Cas9.

In addition, we characterize the influence of decreasing homology arm length on the efficiency of in-trans paired nicking that is applied to fluorescently tag SEC61B at its N-terminus. Next, we also demonstrate that larger knock-ins (almost double in size, i.e. from mScarlet's 750bp to mScarlet-P2A-puro's 1400bp) can be achieved with comparable efficiency, as well as in knock-ins in normal organoids. Although the relative fraction of knock-in cells appears to be lower in tumor organoids, it definitely demonstrates the applicability of ITPN on normal organoids. Moreover, in support of the latter statements is the fluorescent knock-in at the *KRT20* locus in normal organoid lines presented in the new S4 Fig.

Regarding hetero or homozygous knock-ins. Due to the lower electroporation efficiency of organoids, hetero and homozygous knock-in populations were less pronounced during flow analysis when compared to the HEK293T data. In addition, differences between heterozygous and homozygous knock-in populations were less defined, perhaps owing to the more differentiated nature of human (tumor) organoids. Therefore, we decided to remove the statement that inferred homozygosity based on the pattern of the scatter plot. Please note, homozygous knock-ins are being generated in organoids as we observed in clone #7 (S3 Fig).

*2. Fig. 3: The locus that were targeted are actively transcribing/constitutively active (LMNA, HIST1H2BC, CDH1…). Can inactive locus be targeted with similar efficiency? For wider application of this protocol, both actively transcribing and inactive genomic regions must be targeted.*

We acknowledge the sentiment put forward by referee #2 that a robust knock-in strategy should be applicable to all types of genes.

To demonstrate that ITPN can be used to generate knock-ins in genes that are not expressed in the targeted cell population, our revised manuscript now includes a knock-in of mScarlet at the *KRT20* locus in normal human colon organoids. *KRT20* is a well-known differentiation marker for mature, post-mitotic intestinal cells. Since FACS-sorted differentiated cells may not efficiently form organoids, antibiotic selection can only be performed for short periods of time. Alternatively, organoids with mosaic expressions patterns can be manually picked. In S4 Fig, we treated the organoids with a short pulse of the antibiotic Blasticidin starting 10 days after electroporation. This resulted in the successful generation of normal human colon *KRT20* knock-in organoids using ITPN.

*“Further, can an inactive locus in the colonic organoids targeted with a fluorescent reporter be turned-on by in vitro culture modification? OR can a florescent knock-in into an active gene be turned-off transiently? This will determine how well the florescence reports the transcriptional activity of the gene.”*

We appreciate the interest of the referee in the relation between transcriptional activity and fluorescent reporters. In agreement with high accuracy of the reporters, we observed that all the fluorescent proteins of our knock-in constructs localize as expected based on the subcellular localization of the tagged protein. Furthermore, knock-ins at the *KRT20* (S4 Fig) and *LGR5* (rebuttal Fig 1) loci displayed heterogeneous labeling patterns within organoids that is in concordance with expected expression patterns of the endogenous gene. More specifically, mature organoids display a heterogenous expression pattern of KRT20-mScarlet, in agreement with *KRT20* expression that is active in a few differentiated cells. In contrast, expression of the stem cell marker *LGR5* was confined to the base of crypt-like regions in organoids with sharp decreasing levels along the cellular differentiation trajectory towards post-mitotic cell types outside of this region.

Moreover, genetic mouse models provided substantial evidence over the years that knock-ins that are integrated under the control of endogenous gene regulatory regions, faithfully copy the expression patterns of the gene-of-interests. This holds true both *in vivo*, as well as in organoid derivatives of these genetic models. There is no reason to assume that this will be different when the knock-ins are generated in organoids using ITPN. Therefore, we think that additional gene perturbation studies regarding gene expression in the organoids are beyond the scope of the current work. ITPN and the palette of targeting vector that we provide mainly concerns an optimized knock-in strategy for the straight-forward generation of fluorescent gene fusions.

3. How does the size of knock-in construct influence on the efficiency of knock-in? Is the efficiency of knock-in at CDH1 locus similar to "mScarlet" (Fig. 3) and "mScarlet-P2A-Blasticidin" (Sup Fig. 1)? How much does insert size influence on the efficiency of knock-in with this strategy in this manuscript?

We agree with the referee that it is important for future users to know whether their desired reporter construct will be compatible with ITPN-mediated integration.

In the revised Fig 1 we now include a side-by-side comparison of a nearly-identical mScarlet (~0.7kb) versus an mScarlet-P2A-Puromycin^R^ (~1.4kb) integration efficiency at the *HIST1H2BC* locus in tumor organoids. We electroporated equal numbers of targeting vector molecules in each editing condition (i.e., equimolar). Intriguingly, we observed no dramatic difference in editing efficiency. Since most fluorescent gene editing applications will be within this range (i.e., <1.4kb being either a fluorescent reporter only, or in combination with an antibiotic resistance cassette), this covers most applications. Please note: as mentioned above regarding our recent LGR5 knock-ins (Rebuttal Fig 1), we have generated multiple types of knock-ins in normal organoids using >3kb of (multipurpose) reporter alleles.

4. Fig. 3 and Sup Fig. 1: How many biological repeats have been conducted for this strategy? How much efficiency variations have been observed?

This is a good remark, as we strive to be as transparent about ITPN targeting efficiencies as possible.

First, all the new organoid-related data concerning targeting efficiency of different strategies are compiled from at least 3 experiments. This data replaced the original HEK293T data in Fig 1. Please note, variation is presented in the graphs using error bars.

Regarding the triple knock-ins that we presented in Fig 3. We took 3 different strategies, now referred to as triple KI-1, -2 and -3, in which we interchanged the third gene target by different candidates. *CDH1* and *HIST1H2BC* were targeted in all three attempts using identical donor templates.

We now included a table in Fig 3 that shows editing efficiencies for all the independent genes in all three strategies. Similarly, as these strategies were multiplexed, we also indicate the fraction of cells (%) of the targeted population that contain double (all combination types) or triple knock-ins. In general, the variation in targeting efficiency is minimal as is best observed between the 3 independent targetings of *CDH1* or *HIST1H2BC*, as well as the fraction of cells containing both of these knock-ins (being mSc+mTq). Moreover, even when a different third candidate gene was included per attempt, overall targeting efficiencies for double or triple knock-ins showed comparable numbers. Most importantly, as we don't want to focus too much on absolute efficiency specifics, we wanted to highlight that multiplexed gene editing is well worth considering as the protocol is robust and efficient enough.

The two-step knock-in strategy in S7 Fig is foremost a showcase that antibiotic resistance cassettes can be used to enrich the targeted cell population to obtain a nearly pure population of positive cells. All three knock-in strategies share the first targeting of *CDH1*, which is nearly 100% because of Blast selection followed by manual picking. Subsequently, this population is targeted for *HIST1H2BC* in combination with either *MAP4*, *SEC61B* or *LMNA* and was selected for *HIST1H2BC* using puromycin. Their independent targeting efficiencies (all N=1) are depicted in the scatter plots. Note, although not directly selected for correct knock-ins in the third gene-of-interest, these efficiencies are heavily influenced by the fact that selection was applied for cells that correctly integrated HIST1H2BC-mTq, increasing the odds that these cells would also integrate mNeongreen at the indicated genes-of-interest.

*5. Have the authors tested whether organoid lines generated by the multiplex fluorescent gene tagging strategy can be stably maintained over multiple passages? Can organoid lines with 3x fluorescent reporters be frozen (liqN2), recovered and subculture for long-term tracing? It would be informative to provide these further validations for wider utility.*

Yes. The original triple knock-in lines have been frozen for storage. We have now recovered them and continued their culture to expand the knock-in lines for genomic analysis. All three fluorescent reporter genes remain visible and the organoids behave as expected. In addition, on multiple occasions in the last few months we have recovered alternative organoid lines that had been modified using in-trans paired nicking. As shown by our new sequencing data of all the knock-ins, the fluorescent reporters are correctly incorporated within the genome and as such stably inherited. Therefore, our data suggest that our knock-ins in human organoids behave similarly to fluorescent knock-ins in mouse models, which are widely validated and known to be robust.

*6. It would be informative to provide the sanger sequencing results for the targeted locus for Fig. 3, Fig. 4, and Sup. Fig. 1.*

In the revised manuscript, we now provide extensive sequence validation of our knock-in clones that were generated in the new Fig 1, as well as in S4 Fig and the multiplex knock-ins of Figs 3 and 4.

*Clarity and context: The manuscript has been well written with a logical flow and a clear abstract, introduction, and conclusion.*

**Reviewer #3** [identifies himself as Andrew Bassett]

Summary

*Bollen et al. demonstrate that large knock-ins in human organoids can be achieved with good efficiency and precision through use of in-trans paired nicking (nicking of both the genomic locus and targeting vector). The combination of a nicked targeting vector and nickase enzyme shows comparable overall rates of editing compared with WT Cas9, highlighting the potential of generating knock-ins without inducing a double-stranded break. The potential of this technique is further demonstrated through the generation of a triple KI in human colon organoids and the integration of different fluorescent markers on each allele at a single locus, the latter of which establishes a method for the study of allele-specific expression dynamics. Finally, to address the lengthy cloning process associated with generating targeting vectors with homology arms, the authors demonstrate a one-step targeting vector assembly strategy utilising Golden-gate cloning, resulting in a targeting vector with the specific upstream and downstream homology regions on either side of a fluorescent tag. The targeting vector backbone variants developed during this work are available through Addgene.*

*I find the paper to be well written, clear and easy to understand, and the supplementary files provide a clear overview of the strategy and combined with the availability of vectors would enable others to use this methodology. Experiments were well thought out and performed, and do show the versatility and precision of in-trans paired nicking over conventional CRISPR-mediated knock-in methods.*

*I would recommend acceptance in the methods section of PlOS Biology once experiments to address on and off target mutations and integrations listed below have been addressed, and the minor comments have been corrected.*

We thank the reviewer for his positive assessment of our work. We are glad to notice that the reviewer highlights the added value of our work to the research community, in particular the efficiency, precision, and versatility of the approach. We acknowledge that we underperformed on the sequence validation of our approach. This issue has now been addressed in the revised manuscript, where we have added a range of additional experiments to validate the fidelity of the editing approach.

Major Issues

*1. As referenced in this manuscript, the technique of in-trans paired nicking has already been established in other systems such as human cells. Thus, the principal novelty of this study is the application of this technique to human organoids*

Indeed, while the editing technique itself is not novel, we believe the novelty of our manuscript is provided by our streamlined protocol and targeting vector palette that is broadly applicable, our extensive confirmation of the fidelity of in-trans paired nicking at several genomic loci and the multiplexed fluorescent gene editing in human organoids.

*2. Integrity of non-targeted allele. One of the major benefits of the in trans paired nicking system is that the non-targeted allele should not accumulate indel mutations, allowing for instance multiple rounds of targeting without sequence validation and cloning in-between and meaning that e.g. "…sequence verification of individually picked clones is no longer required since all knock-in cells can immediately be pooled." However, the authors do not directly demonstrate this precision. They should perform experiments to address the frequency of mutations at the non-targeted allele for instance by high throughput sequencing of the WT alleles after a targeting experiment in comparison to the DSB-mediated methodologies.*

We appreciate the valuable comments regarding sequence verification of knock-in integrity. These prompted us to perform the necessary control experiments that in our view resulted in a much stronger manuscript.

In our revised manuscript we now include extensive confirmation regarding the fidelity of in-trans paired nicking. With respect to the integrity of the non-targeted allele:

We now show TIDE analyses of the non-targeted alleles in the *SEC61B* knock-in lines of Fig 1 that are generated by either ITPC (wild-type Cas9) or ITPN (nickase). As expected, when editing using Cas9 nickase, the integrity of the wild-type allele is preserved as mutations were not reliably detected within the bulk knock-in population. In contrast, editing using wild-type Cas9 resulted in a fraction of organoids with mutations in the non-targeted allele in all assessed cases. This data is now shown in S2 Fig.

Next, from the ITPN knock-in condition we isolated and expanded 11 clonal lines carrying the mScarlet knock-in at the N-terminus of the *SEC61B* locus. In these lines we again confirmed the absence of indels in the wild-type allele for heterozygous clones. This data is now shown in S3 Fig.

Using TIDE analysis, we also confirmed the absence of mutations in the non-targeted alleles of the genes targeted in the triple knock-in lines shown in Fig 3. This data is now shown in S5 Fig.

*3. On target precision. Similarly, it has been shown that DSB-mediated genome engineering can introduce undesired and unexpected mutations on target such as long deletions (Kosicki et al. NatBiotech 2018, "Repair of double-strand breaks induced by CRISPR-Cas9 leads to large deletions and complex rearrangements"), and tandem integrations of multiple copies of the transgene, etc (Canaj et al. BioRxIV 2019 "Deep profiling reveals substantial heterogeneity of integration outcomes in CRISPR knock-in experiments"). It would be important to assess the frequency of these on target mutations with the different strategies. This could be done by targeted long read sequencing (e.g. ONT), or long-range PCR and Sanger sequencing.*

From the 11 clonal lines that we generated from the ITPN-mediated mScarlet knock-in at the N-terminus of *SEC61B*, we now confirmed perfect integrity of the 5' and 3' junctions of the integrated reporter in all lines using PCR amplification of the knock-in followed by Sanger sequencing. Moreover, the PCR amplified knock-in bands were of correct size, indicating that tandem integrations and long deletions have not occurred. This data is now shown in the new S3 Fig.

Next, for the triple knock-in lines in Fig 3 we also confirmed the integrity of the upstream and downstream junction of the knock-in. This data is now shown in S6 Fig.

In addition, we also confirm the integrity of the junctions for the bi-allelic knock-ins (i.e., 5' and 3' end of both reporter knock-ins for 3 genes) presented in Fig 4. This data is now shown in S8 Fig.

*4. Off-target mutation. Another advantage of using Cas9 nickase is the reduction of off-target mutations. I feel that this is not addressed experimentally and should be done through sequencing potential off-target sites (in comparison to nucleases). A key reference that should be included on this topic is Chen et al. NAR 2020 "Expanding the editable genome and CRISPR-Cas9 versatility using DNA cutting-free gene targeting based on in trans paired nicking."*

In the revision experiments we have found no evidence for the presence of indels in the non-targeted allele. Given that this allele serves as a proxy for the highest ranking 'off-target' candidate through its perfect sequence similarity to the gRNA, we interpret these data as a confirmation that the chance for indel mutations at alternative genomic loci where the gRNA has mismatches is highly unlikely.

To fully address the advantage of Cas9 nickases in terms of reducing off-target mutations, we have analyzed the genomes of three knock-in clones via whole-genome sequencing. No mutations were detected at 166 in silico predicted potential off-target sites.

*5. Off-target integration. dsDNA molecules, especially when linearized, are able to integrate randomly (as well as at CRISPR off-target sites) in the genome. The authors should address the frequency at which this occurs by performing analysis of random integration of transgenes (e.g. by ddPCR copy number counting or inverse PCR).*

We appreciate the reviewer's interest regarding dsDNA integration into off-target CRISPR sites once linearized. However, we emphasize that, in contrast to ITPC, there are no linearized plasmids when using nickases and the chance for off-target integration is thereby minimized.

Nevertheless, we analyzed the three ITPN-mediated knock-in clones that were whole-genome sequenced for random integration of nicked donor templates, or any part of the used targeting vector plasmids and found no evidence for this to happen. Moreover, as described earlier to point 3, in the 11 clonal lines that we established we found no evidence for tandem integration at the target sites.

Minor Issues

*1. "By contrast, flanking homology arms with Cas9 target sites to facilitate in-trans paired cleavage saturates the homology demand at around ~600bp [10]" I'm not sure the paper they cite really demonstrates this difference, since it seems locus dependent (for instance, Fig 2C is quite similar between the paired cleavage and circular donors). They should adapt the text accordingly*

We agree with the referee, the manuscript has been edited accordingly.

*2. The authors should cite additional papers describing the in trans paired nicking approach such as (but not restricted to) the following:*

*a. Rees et al. 2019 NatComms "Expanding the editable genome and CRISPR-Cas9 versatility using DNA cutting-free gene targeting based on in trans paired nicking " that uses a Cas9 nickase fusion to Rad51 to improve editing outcomes.*

*b. Hyodo et al. 2020 CellRep "Tandem Paired Nicking Promotes Precise Genome Editing with Scarce Interference by p53"*

We thank the reviewer for these suggestions. Citations to these additional papers have now been included. However, since we prefer editing using a single Cas9 guide, we mention Hyodo et al. solely as an example of Cas9 nickase mediated editing.

*3. Figure 1 - 5' and 3' annotation on DNA incorrect.*

We thank the reviewer for drawing our attention to this error. We have corrected it in the revised manuscript.

*4. Supplementary Figure 1 - Incorrect spelling of 'triple' in diagram title.*

This has now been corrected in the revised manuscript.
